# Supplementary material for: Assessment of antibody dynamics and neutralizing activity using serological assay after SARS-CoV-2 infection and vaccination
Source: PLoS One. 2023 Sep 19;18(9):e0291670. doi: 10.1371/journal.pone.0291670 (PMC10508622; doi:10.1371/journal.pone.0291670)
Supplement: Attachment — Submitted filename: Response to Reviewers .docx [file pone.0291670.s001.docx]

August 15th, 2023

Dr. Etsuro Ito

Academic Editor

PLOS ONE

RE: Resubmission of the revised manuscript MS# PONE-D-22-35551R1 titled “Assessment of antibody dynamics and neutralizing activity using serological assay after SARS-CoV-2 infection and vaccination” by Takahashi T., et al.

Dear Dr. Etsuro Ito,

We appreciate the reviewers for their highly constructive comments for our manuscript, and we believe that we fully addressed the issues as detailed below.

Reply (Answer; A) to Reviewer’s comments and questions (Comment; C):

**Reviewer 1**

**COMMENTS by Reviewer #1:**

The authors showed that antibody dynamics and neutralizing activity following COVID-19 vaccination/ infection among patients, HCWs. Although the study poluation and background seems unique, there're some points to be revised. And the manuscript is redundant with too many figures. Simplify is also required.

**C1**: The PECO of the study is unclear. 'Investigate the serological kinetics' seems to be vague.

**A1**: We appreciate the reviewer’s suggestion. Following the comment, we rephrased the sentence. (L74-77)

**C2**: In Line 105, should clarify the number of community acquired/ nosocomial COVID-19 case. In Line 106, should describe the breakdown of HCWs (i.e., Nurses N=, doctors N=, Lab technician N= ...)

**A2**: Following the reviewer’s suggestion, we added the ~~detail~~ number of patients and HCWs. (L92,94)

**C3**:

a. The first half of result is overlapped with Table1. Table 1 should be deleted. Or simplify the manuscript including quotation of Table1.
b. For Figure 2A, Plot of each group (S and M) should combined.

For Figure 2B, Plot of each group (S and M) should combined.

c. Number of the categolized study population shoud be noted on the figure. (i.e., number of the subject in N-IgG pisitive & COVID-19 Medical History positive in Figure 3A.)
d. Most of figure legends are overlapped with each figures. Delete overlapped description in the manuscript.

**A3**: Thank you for the valuable comments. Following the reviewer’s suggestion, we changed as follows.

a. Table1 has been deleted.
b. We combined the plot of each group (S and M) and changed the legend accordingly in Figure 2A and B.
c. We added the number of the categorized study population in Figure 3A.
d. We deleted the description in the manuscript and figure which were overlapped with figure legends for Figure 1 and 2.

**C4**: The authors add the thought, or plan, of potential for clinical use in real world according to the result of the study.

**A4**: We appreciate the reviewer's suggestion. A description of the potential for medical epidemiological use in the real world has been added. (L307-313)

**Reviewer 2**

COMMENTS by Reviewer #2:

This is an important study to compare the three different antibody analysis methods and their kinetics among COVID-19 cases and HCWs after 2-dose vaccination, using authentic virus neutralization assay as a reference. Please consider to revise the manuscript, based on the comments listed below.

**C1**: L 47-49: Does this sentence mean that “low titer of” anti-S IgG or sVNT causes breakthrough infection? The meaning is a little bit difficult to understand.

Overall, please consider to revise the abstract accordant with the revision of the main text, tables and figures.

**A1**: We apologize for the confusion caused by the previous explanation. We described that it is difficult to determine whether an antibody titer of anti-S IgG or sVNT is causing a breakthrough infection (L46-47). The overall correction has been made to reflect other comments.

**C2**: The aim of this study is not matched with the conclusion. Please revise in accordance with the other contents of the manuscript.

**A2**: Following the reviewer’s suggestion, the description of the aim has been changed. (Abstract, L29-38; introduction, L74-77)

**C3**:

a. L 109-110: Immunosuppressive agents are commonly used to treat COVID-19 itself. Does this study exclude COVID-19 patients treated with immunosuppressive agents such as corticosteroid or other biologic agents? Or are the patients using immunosuppressive agents for their underlying diseases before contracting COVID-19 excluded ?
b. It is unclear how the authors collected the history of contracting COVID-19 from the HCWs and how they identified infection with delta variant or other variants.
c. L120: Please describe the detailed material and methods of the long-term evaluation study.
d. L170: The meaning of “When experiments involved more than two groups” is difficult to understand.
e. L175: What is the longitudinal analysis? Is it same as “long-term evaluation study”? Please clarify the meaning.

**A3**:

a. We apologize for any confusion caused by the previous description. We clarified that in this study, we excluded patients who had received immunosuppressive drugs for their underlying disease prior to contracting COVID-19 (L101-102).
b. We apologize for the inadequate description. We described how we identified the past history of COVID-19 infection in HCWs and identified the type of variants (L95-96, L98-101).
c. We described the analytical method of the longitudinal assessment study in the Statistical analysis section in “Material and Methods” (L167-169).
d. We apologize for the unclear wording. We corrected the sentences. (L162-164)
e. We reworded “long-term evaluation study” to “longitudinal assessment study” (L112,167).

**C4**:

a. Overall, description about the methods seems mixed in the Result part. For example, L182-183, L214-221, L240-245.
b. L269-272: As pointed out in the Material and Methods part, please clarify the methods to identify the past history of COVID-19 of HCWs and type of variants and revise the description if necessary.

**A4**:

a. Thank you for bringing this to our attention. We apologize for the mix up. Following the reviewer’s suggestion, we have deleted the duplicate text in the "Results" section.
b. Please see **A3b**.

**C5**:

a. L294-296: Relatedness with the former sentences in L292-294 is a little bit confusing. Do the authors intend to say the authentic virus neutralization activity increases more slowly/weakly than anti-S antibody and/or sVNT?
b. L328: the distribution of age in the Groups should be written in the Result part.
c. L331-333: The conclusion is not matched with the aim of the study described at the end of the Introduction part (investigation or serologic kinetics).
d. The authors conclude that usefulness of titers of anti-S IgG and sVNT as surrogate markers of neutralizing capacity is limited, but as an experiment, concordance of titers of anti-S IgG and sVNT with authentic neutralization assay seems acceptable, even though some cases show discrepancy. Do the authors intend to describe the insufficiency of the three markers to predict the ability to prevent infection?

**A5**:

a. Thank you for your ~~helpful~~ comment. We rephased the sentence. (L272-274)
b. Following the reviewer’s suggestion, the age distribution was noted in the "Results" section and the statement of the discussion was changed. (L203-204, L304-305)
c. We agree with the reviewer’s comment and changed the statements of the aim and the conclusion. Please see **A2**.
d. Yes, we conclude that the use of anti-S IgG titers as a direct quantitative marker of neutralizing capacity has limitation and that serological tests should be interpreted with caution in the treatment of COVID-19. On the other hand, we also noted that quantitative S-IgG measurements suggest that they can be used in epidemiological studies to provide important information for future control of SARS-CoV-2 infection. (L307-313)

**COMMENTS (continued):**

The authors might consider some minor comments:

**C1**: L114: SpO2 should be written with small "2".

L133: AU/ml should be written as AU/mL.

**A1**: Corrected.
